# *β*-glucans, SAM, and GSH fluctuations in barley anther tissue culture conditions affect regenerants’ DNA methylation and GPRE

**DOI:** 10.1186/s12870-024-05572-w

**Published:** 2024-09-12

**Authors:** Renata Orłowska, Wioletta Monika Dynkowska, Agnieszka Niedziela, Jacek Zebrowski, Janusz Zimny, Piotr Androsiuk, Piotr Tomasz Bednarek

**Affiliations:** 1 Plant Breeding and Acclimatization Institute-National Research Institute, Radzików, 05-870 Błonie Poland; 2 Institute of Biology and Biotechnology, University of Rzeszow, Al. Rejtana 16C, Rzeszow, 35-959 Poland; 3 Department of Plant Physiology, Genetics and Biotechnology, Faculty of Biology and Biotechnology, University of Warmia and Mazury in Olsztyn, Olsztyn, 10-719 Poland

**Keywords:** Anther culture, Barley, *β*-glucans, GSH, SAM, DNA methylation, DNA sequence, Green plant regeneration efficiency

## Abstract

**Background:**

Microspore embryogenesis is a process that produces doubled haploids in tissue culture environments and is widely used in cereal plants. The efficient production of green regenerants requires stresses that could be sensed at the level of glycolysis, followed by the Krebs cycle and electron transfer chain. The latter can be affected by Cu(II) ion concentration in the induction media acting as cofactors of biochemical reactions, indirectly influencing the production of glutathione (GSH) and S-adenosyl-L-methionine (SAM) and thereby affecting epigenetic mechanisms involving DNA methylation (demethylation—DM, *de novo* methylation—DNM). The conclusions mentioned were acquired from research on triticale regenerants, but there is no similar research on barley. In this way, the study looks at how DNM, DM, Cu(II), SAM, GSH, and *β*-glucan affect the ability of green plant regeneration efficiency (GPRE).

**Results:**

The experiment involved spring barley regenerants obtained through anther culture. Nine variants (trials) of induction media were created by adding copper (CuSO_4_: 0.1; 5; 10 µM) and silver salts (AgNO_3_: 0; 10; 60 µM), with varying incubation times for the anthers (21, 28, and 35 days). Changes in DNA methylation were estimated using the DArTseqMet molecular marker method, which also detects cytosine methylation.

Phenotype variability in *β*-glucans, SAM and GSH induced by the nutrient treatments was assessed using tentative assignments based on the Attenuated Total Reflectance-Fourier Transform Infrared (ATR-FTIR) spectroscopy. The effectiveness of green plant regeneration ranged from 0.1 to 2.91 plants per 100 plated anthers. The level of demethylation ranged from 7.61 to 32.29, while *de novo* methylation reached values ranging from 6.83 to 32.27. The paper demonstrates that the samples from specific *in vitro* conditions (trials) formed tight groups linked to the factors contributing to the two main components responsible for 55.05% of the variance (to the first component DNM, DM, to the second component GSH, *β*-glucans, Cu(II), GPRE).

**Conclusions:**

We can conclude that *in vitro* tissue culture conditions affect biochemical levels, DNA methylation changes, and GPRE. Increasing Cu(II) concentration in the IM impacts the metabolism and DNA methylation, elevating GPRE. Thus, changing Cu(II) concentration in the IM is fair to expect to boost GPRE.

## Background

Barley (*Hordeum vulgare* L.) is a cereal that has been economically important for centuries. It originated from the wild form *Hordeum vulgare* ssp. spontaneum and was probably domesticated in the Middle East around 8000 BC [1]. Today, barley continues to play a significant role in feeding [2], brewing [3], and distilling [4]. It is also a model plant in many plant studies, including those that employ double haploids (DH). The generation by tissue culture of plants with a completely homozygous doubled chromosome set in a single cycle eliminates the expenditure of time and labour. It is, therefore, so desirable for breeding work. Haploid forms have a different morphology from diploids; they are smaller than diploid barley, have thinner leaves, are more bushy, and their gametes are mostly aborted. Therefore, they are not the primary target for breeding and are usually eliminated during research or breeding processes. Thus, doubling the number of chromosomes during development is necessary for a chance of seed set and reproductive success.

Barley plants can be grown as doubled haploids using the "bulbosum" method [5] or through embryogenesis of microspores in anther [6] or isolated microspore cultures [7]. The isolated microspore method has been around for over thirty years, whereas the "bulbosum" and other methods for almost fifty years. These techniques are designed to generate homozygous, homogenous materials fit for breeding purposes [8, 9] or fundamental research [10].

Using anther or isolated microspore cultures to grow plants does not necessarily result in homogeneous plants because of changes to the tissue culture or differences already present in the explants (pre-existing variation) [11]. It is crucial to reduce such variation to the lowest level possible if the impact of the *in vitro* culture is to be studied. According to studies of anther culture triticale and barley regenerants that underwent a generative cycle [12–14], it is possible to control the phenomenon using the generative offspring of a self-evolved single regenerant that was a doubled haploid [12–14].

However, genetic or epigenetic variation may impact the uniformity of regenerants produced by tissue culture. Such regenerants are hampered by tissue culture-induced variation (TCIV) [15, 16], whereas their generative progeny are by somaclonal variation [12, 17–19]. TCIV refers to changes arising at the DNA sequence level and in the DNA methylation of the regenerants. Hence, the studies presented here describe epigenetic phenomena concerning plant tissue cultures. In addition to DNA methylation, several other mechanisms, such as histone methylation, chromatin remodelling, and short RNA, belong to the epigenetic aspects (described in the review papers) [20–23].

Various methods are used to assess tissue culture-induced variation and rely on different forms of DNA markers. Grass species have been studied using both dominant (Inter-Simple Sequence Repeat: ISSR, Random Amplified Polymorphic DNA: RAPD, Amplified Fragment Length Polymorphism: AFLP, Inter-Retrotransposon Amplified Polymorphism: IRAP) [24–26] and codominant (Restriction Fragment Length Polymorphism: RFLP, Simple Sequence Repeats: SSR) marker techniques [27, 28]. These marker techniques primarily investigate the genetic nature of TCIV variation, whereas RFLP (methylation-sensitive RFLP) [29, 30], AFLP (metAFLP) [31], and the Methylation-Sensitive Amplified Polymorphism (MSAP) [32] techniques could be employed to study the methylation aspect. Some of these methods were also used to assess TCIV in triticale [33] and barley [34]. An alternative option is to use a new-generation sequencing strategy. The MSAP quantitative method was employed in the Diversity Arrays Technology Methylation Analysis (DArTseqMet) approach [35] to explore the TCIV shared by regenerants from barley cultures. The methodology focused on improving the effectiveness of green plant regeneration [35]. Cu(II) ion concentration in the induction media (IM) influences GPRE, as was shown in triticale [36]. In earlier investigations, Ag(I)'s function and timing of a subsequent culture were also discussed [35].

Variation induced in plant tissue cultures also includes changes that may involve the regenerant metabolome [37]. The Attenuated Total Reflectance (ATR) technique can investigate biochemical changes by directly analysing solid or liquid samples without additional preparation [38]. The ATR technique is commonly combined with Fourier Transformed Infrared (FTIR) spectroscopy (ATR-FTIR) to simplify the measurement. The ease of preparation and the small sample size that can be recovered from the crystal surface if necessary [39] are undeniable advantages of ATR-FTIR. This method has been used extensively to study plant materials considering different growth conditions and abiotic stresses affecting the biochemical phenotype [40–43] and in studies on barley [34] and triticale [37] plants derived via androgenesis in anther culture.

The success of green plant regeneration by androgenesis is influenced by many factors, ranging from the appropriate treatment of harvested tillers with spikes (low temperature [44], darkness [45], glutathione supplementation [46]) to the modification of tissue culture media with plant growth regulators [47], sugars [48], micro- and macronutrients [49]. Recent studies on the regeneration of barley and triticale by androgenesis in anther cultures have revealed relationships between TCIV, supplementation of induction media with Cu(II) ions and green plant regeneration efficiency (GPRE). In addition, metabolites such as glutathione (GSH) [50], S-adenosyl-L-methionine (SAM) [51], and *β*-glucan [34] were identified as those that could be affected by ions (Cu(II), Ag(I)) added to induction media through metabolic pathways.

*β*-glucans are the primary substrates for glycolysis, pumping the Krebs cycle [52]. The latter is linked to the electron transfer chain (ETC), resulting in ATP. SAM is made when ATP and S-adenosyl-L-methionine are combined (the Yang cycle). SAM is the leading methylating agent in cells [53] and is in charge of more than 80% of methylation events, such as DNA methylation. Moreover, via the transsulfuration pathway, the Yang cycle indirectly affects GSH synthesis [53, 54]. The ETC depends on Cu(II). Thus, any imbalances in the ion concentration should affect ATP production and the synthesis of the following metabolites.

GPRE is promoted by Cu(II) ion in the IM acting as cofactors of biochemical reactions, including those of the electron transport chain (ETC), the Yang cycle and GSH synthesis pathways affecting epigenetic mechanisms involving DNA methylation. Such a hypothesis was tested on triticale and is believed to be a more general mechanism in anther cultures of cereals. We hypothesize that ions acting as cofactors may differently affect tissue cultures, inducing varying stresses influencing biochemical reactions that could be studied via metabolome fluctuations. Furthermore, fluctuations may affect metabolome and, thus, GPRE. As such, subtle changes should be linked, and understanding the relationships between different levels of cells functioning during androgenesis is crucial for future progress in DH production. The study identifies relationships between DNM, DM, Cu(II), SAM, GSH and *β*-glucan influencing GPRE.

## Methods

### Plant material

The study used spring barley (*Hordeum vulgare* L.) genotype NAD2 from Poznan Plant Breeding Ltd. (Nagradowice, Poland). Donor plants that arose by self-pollination of androgenic regenerant (doubled haploid) were selected based on their morphology, including plant height, leaf shape, and type of tillering. Anther cultures were established using explants (anthers) from the donor plants to obtain regenerants through androgenesis. The donors were prepared for analysis as described previously [16].

The process for obtaining regenerants was previously outlined elsewhere [55]. The necessary steps are as follows: tillers from donor plants were harvested 6–10 weeks after being planted into pots and cut when microspores were in the mid-to-uninucleate stage. Cut tillers with spikes were stored in plastic bags in the dark at 4 °C for 21 days. After this period, anthers were plated on N6L induction media (IM) [56] supplemented with 2 mg l^−1^ 2,4-D (2,4-dichlorophenoxyacetic acid), 0.5 mg l^−1^ NAA (naphthaleneacetic acid), and 0.5 mg l^−1^ kinetin and incubated in the dark at 26 °C. The IM also contained varying concentrations of salts, such as CuSO_4_ (copper sulfate: 0.1; 5; 10 µM) and AgNO_3_ (silver nitrate: 0; 10; 60 µM). Different incubation times (21, 28, and 35 days) were used for the anther cultures, and nine IM (A-I) variants were prepared. The incubation time of the explants on the induction media covers points from the plating anthers on IM to calli, embryo-like structures, and embryo collection and transfers them onto regeneration media. The next step was regeneration on K4NB media [57] containing 0.225 mg l^−1^ BAP (6-benzylaminopurine). The regeneration process occurred at 26 °C during the 16-h day and 8-h night. The rooting of the regenerated green plants took place on N6I medium [56] supplemented with 2 mg l^−1^ IAA (indole-3-acetic acid). The resulting plants were transferred to pots and grown in a greenhouse (16 h light, 16 °C / 8 h dark, 12 °C) until maturation. The morphology of green regenerants was assessed based on traits such as plant height, leaf shape, type of tillering, and seed-setting ability, which allowed for the estimation of the spontaneous doubling of the number of chromosomes. Finally, GPRE was evaluated based on the number of green regenerants produced per 100 plated anthers. Thirty-five plants derived from a single donor plant, obtained in all variants tested (A-I), were selected for analysis.

### DNA extraction, DArTseqMet genotyping and quantification of variation

We used a commercial kit called DNeasy MiniPrep from Qiagen (Hilden, Germany) to extract DNA. Leaves from the donor plant and regenerants were collected during the tillering stage. We assessed the quantity of the DNA samples using spectrophotometry and checked their quality by running them through a 2% agarose gel with ethidium bromide.

DArT PL (https://www.diversityarrays.com) performed DArTseqMet on 35 regenerants. The resulting markers were converted into semi-quantitative methylation characteristics using the MSAP technique. The conversion involved the analysis of molecular data produced by HpaII and MspI endonucleases, interpreting the results to identify specific events, quantifying them, and developing MSAP characteristics that indicate changes in DNA methylation (such as demethylation (DM) or *de novo* methylation (DNM)) [58].

### ATR-FTIR

The samples were first lyophilized using a laboratory freeze dryer (Alpha 1–4 LSC Christ, Polygen, Østerode, Germany) and then homogenized into powder using a ball mill (MM 400, Retsch, Haan, Germany) to conduct mid-infrared spectroscopy. The measurements were taken using the iZ10 module of the Nicolet iN10 MX infrared imaging microscope (Thermo Fisher Scientific, Waltham, MA, USA). The microscope was equipped with a deuterated triglycine sulfate (DTGS) detector and a KBr beam splitter. Sixty-four spectra were collected per sample in the Attenuated Total Reflectance (ATR) mode at 4 cm^−1^ resolution in the wavenumber range between 600 and 4000 cm^−1^. The measurements were taken using the one-bounce diamond crystal and the ATR accessory (Smart Orbit, Thermo Scientific, Madison, WI, USA). The surface of the diamond crystal was cleaned with water or propanol before each measurement to remove residuals from previous samples. The spectra were recorded, averaged, and baseline-corrected using OMNIC software (v.9.0, Thermo Fischer Scientific Inc.). The 1^st^ derivative was calculated and vector normalized following Savitzky-Golay filtering and smoothing. The calculations, statistics (mean, SD) and plots of spectra were performed using the ChemoSpec package [59] (functions: normSpectra and surveySpectra (method = ″sd″)) in the R programming language [60].

### Statistical analyses

Pearson’s correlation analysis, Principal Component Analysis, Classification & Regression Trees, and the Passing-Bablok regression were conducted in XlStat 2020.1.1 Excel add-inn [61].

## Results

A double haploid barley plant produced progeny consistent in height, leaf size, tillering, and seed set. Explant tissue was taken from one of the progeny plants. The concentration of Cu(II) and Ag(I) ions in the IM, as well as the time of anther cultures, were altered in nine trials (A-I). Finally, 35 regenerants entirely in type as donor plants regarding plant morphology were derived. No other (off-type) regenerants were evaluated. In each trial, there were between 3 and 5 regenerants. According to Table 1, the effectiveness of green plant regeneration ranged from 0.1 (trial E) to 2.91 (trial G) (Table 1).

**Table 1:** The *in vitro* tissue culture conditions including Cu(II) and Ag(I) ion concentrations in the induction medium (IM), time (days) of anther culture, *β*-glucans, S-adenosyl-L-methionine (SAM) and glutathione (GSH) based on the 1^sd^ derivative of the FTIR spectrum within 990–950, 1630–1620 and 2550–2540 cm^−1^ range, respectively, and efficiency of green plant regeneration (GPRE) as a number of green regenerants obtained by 100 plated anthers contraposed with experimental trials (A-I) and the DArTseqMet quantitative characteristics concerning asymmetric *de novo* DNA methylation (DNM) and DNA demethylation (DM) variations

Trial	*In vitro* tissue culture conditions			DArTseqMet (%)		FTIR data			GPRE
	Cu(II)	Ag(I)	Time	DNM	DM	SAM	GSH	*β*-glucans	
A	0.1	0	21	28.15	32.18	0.37877002	0.037779	0.0443	0.64
A	0.1	0	21	28.19	32.29	0.38572509	0.027657	0.0267	0.64
A	0.1	0	21	28.16	32.25	0.38024021	0.033741	0.0371	0.64
A	0.1	0	21	28.14	32.17	0.36916384	0.029796	0.0317	0.64
A	0.1	0	21	28.1	32.21	0.37316889	0.03685	0.0388	0.64
B	0.1	10	28	31.09	29.43	0.37212605	0.036056	0.0283	0.67
B	0.1	10	28	31.1	29.58	0.37036069	0.028056	0.0287	0.67
B	0.1	10	28	31.01	29.46	0.39685106	0.037014	0.0322	0.67
C	0.1	60	35	30.92	29.29	0.37441652	0.029443	0.0274	1.09
C	0.1	60	35	30.86	29.3	0.38539155	0.029008	0.0264	1.09
C	0.1	60	35	30.88	29.33	0.33857775	0.040079	0.0454	1.09
C	0.1	60	35	30.92	29.28	0.38772455	0.033056	0.0263	1.09
C	0.1	60	35	30.92	29.31	0.40502951	0.037433	0.0255	1.09
D	5	60	28	30.42	30.53	0.37808317	0.030979	0.0282	0.45
D	5	60	28	30.46	30.59	0.38031144	0.03189	0.0325	0.45
E	5	0	35	6.9	7.83	0.36384070	0.029989	0.0329	0.1
E	5	0	35	10.8	11.34	0.35114625	0.043758	0.0484	0.1
E	5	0	35	6.83	7.61	0.34510472	0.043573	0.0483	0.1
E	5	0	35	11.04	11.17	0.37218174	0.032762	0.0294	0.1
F	5	10	21	31.52	28.67	0.38049843	0.037839	0.0353	2.12
F	5	10	21	31.3	28.65	0.37682563	0.037169	0.0343	2.12
F	5	10	21	31.51	28.57	0.37245534	0.030541	0.0238	2.12
F	5	10	21	31.52	28.67	0.37727839	0.034857	0.0311	2.12
F	5	10	21	31.36	28.82	0.37400542	0.038586	0.0331	2.12
G	10	10	35	29.91	29.97	0.39753918	0.034812	0.0268	2.91
G	10	10	35	30	30.11	0.33243911	0.041996	0.0482	2.91
G	10	10	35	29.98	29.99	0.38319139	0.049887	0.0373	2.91
H	10	60	21	31.26	29.66	0.38747164	0.038674	0.044	1.77
H	10	60	21	31.28	29.78	0.35703710	0.036527	0.0533	1.77
H	10	60	21	31.25	29.79	0.36122281	0.03497	0.0426	1.77
I	10	0	28	32.2	28.13	0.41650493	0.043391	0.0441	0.54
I	10	0	28	32.27	28.28	0.38986374	0.038744	0.0361	0.54
I	10	0	28	32.23	28.23	0.37705811	0.040217	0.0388	0.54
I	10	0	28	32.2	28.31	0.39947962	0.03587	0.0335	0.54
I	10	0	28	32.19	28.29	0.39081405	0.02932	0.0223	0.54

DNA quantity and quality from the donor plant's and its regenerants' fourteen-day-old leaves were sufficient for the DArTseqMet analysis. DArTseqMet approach found that the overall DNM and DM ranged from 6.83 to 32.27 and 7.61 to 32.29 (Table 1), respectively, based on the banding patterns shared by the donor plant and its regenerants.

**Fig. 1:**
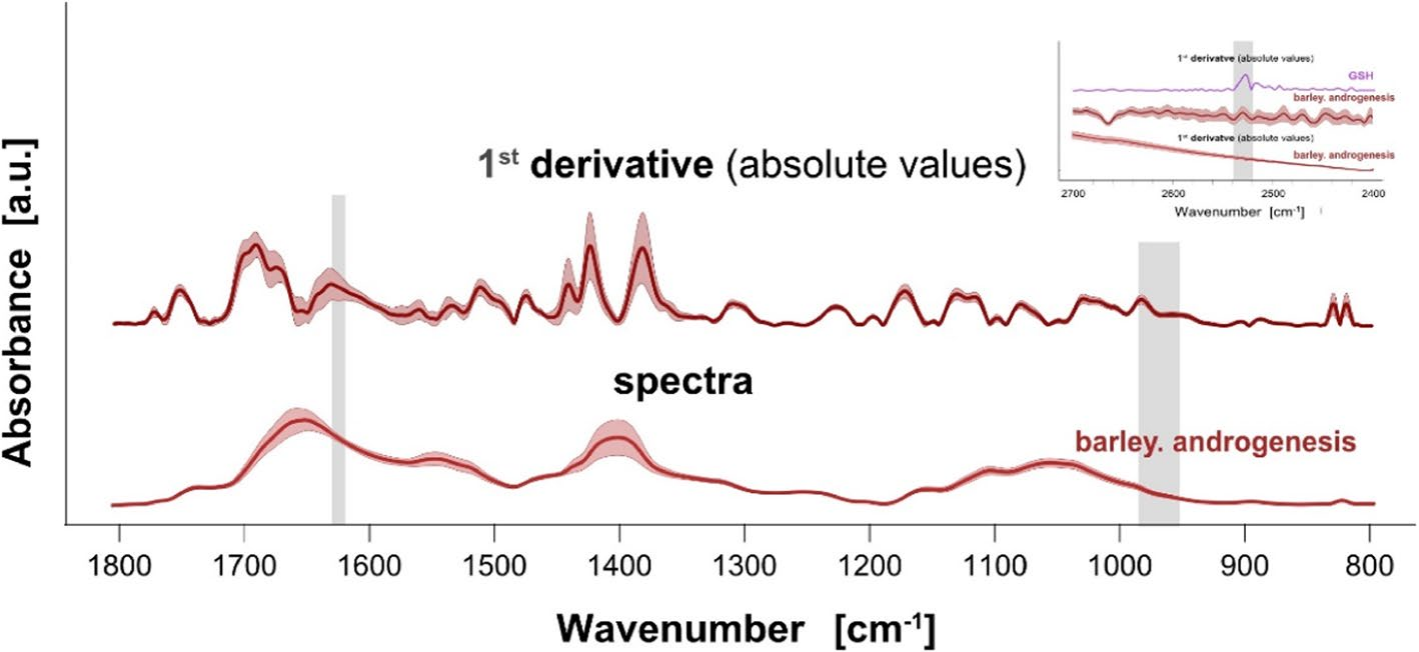
The mean (central line) and the standard deviation (ribbon) of the spectra absorbance (bottom) and the absolute values of calculated the 1^st^ derivative (top) collected from the barley leaves of young regenerants derived via androgenesis. The inset presents the spectra and the 1^st^ derivative for region around 2500 cm^−1^, which can be tentatively assigned to GSH (shaded). The wavenumber ranges corresponding to SAM (1630–1620 cm^−1^) and *β*-glucans (990–950 cm^-1^), are marked grey shaded

The absolute values of the 1^st^ derivative calculated for ATR-FTIR absorbances was used as the numerical input to the analysis. The contribution of compounds under consideration, i.e. *β*-glucans, S-adenosyl-L-methionine and glutathione, was evaluated assuming spectral regions selected in our previous studies [34, 37, 51]. Figure 1 presents collected spectra and absolute values of the 1^st^ derivative calculated on the spectra absorbance, along with shaded regions depicting contribution from *β*-glucans, S-adenosyl-L-methionine (SAM) and glutathione (GSH) at 990–950 cm^−1^, 1630–1620 cm^−1^ and 2550–2540 cm^−1^, respectively.

Bartlett’s sphericity test was significant (*p* < 0.0001 at α < 0.05), indicating that at least one of the correlations (Table 2) between the variables is meaningly different from zero. The highest and positive correlation values were evaluated between DM and DNM, *β*-glucans and GSH. A negative correlation was observed between *β*-glucans and SAM. The remaining significant correlations were both positive and negative but below 0.5.

**Table 2:** Pearson’s correlation matrix—analysis between original results reflecting Cu(II) and Ag(I) ion concentrations, time of anther culture, percentages of DNM and DM, FTIR spectra assigned to SAM, GSH, and *β*-glucans, and GPRE at *p* < 0.05

Variables	Cu(II)	Ag(I)	Time	DNM	DM	SAM	GSH	*β-*glucans	GPRE
Cu(II)	**1**								
Ag(I)	-0.107	**1**							
Time	-0.002	0.160	**1**						
DNM	0.065	0.307	**-0.400**	**1**					
DM	-0.106	0.269	**-0.472**	**0.939**	**1**				
SAM	0.058	-0.048	-0.118	**0.412**	**0.346**	**1**			
GSH	**0.407**	-0.179	0.176	-0.092	-0.152	-0.140	**1**		
*β*-glucans	**0.337**	-0.017	-0.079	-0.224	-0.194	**-0.505**	**0.679**	**1**	
GPRE	**0.345**	0.156	-0.138	**0.444**	**0.400**	-0.067	0.266	0.049	**1**

Values in bold are different from 0 with a significance level alpha = 0.05

The Principal Component analysis eigenvalues have shown that the first three components explain nearly 68.7% of the variance (Table 3).

**Table 3:** The eigenvalues of the principal component analysis

	F1	F2	F3
Eigenvalue	2.839	2.118	1.229
Variability (%)	31.542	23.534	13.661
Cumulative %	31.542	55.076	68.737

According to the PCA, the samples representing trials form more or less uniform clusters. For example, see samples of trials H, I, E and C. Some samples of different trials overlap (A, B, C, and D).

**Fig. 2:**
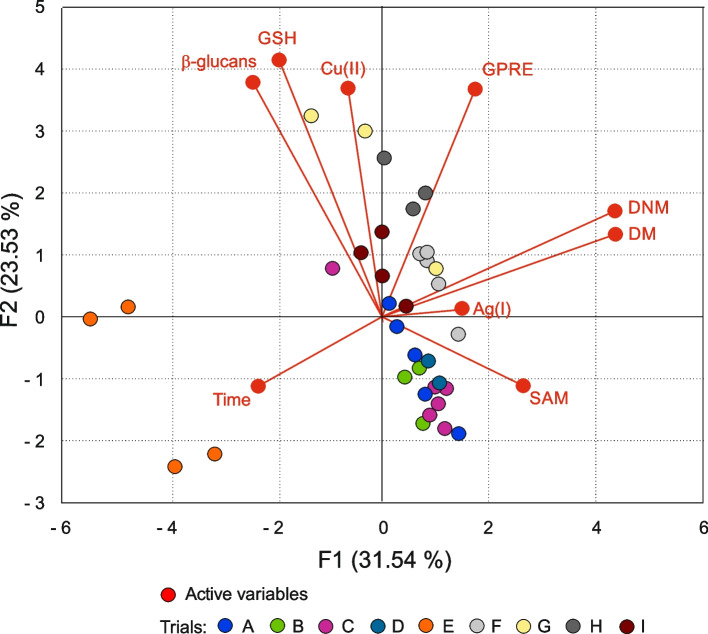
Principal component analysis biplot. Active variables (for explanation see Table 1) are given in red whereas trials are indicated in colored dots

The factor loadings (Table 4, Fig. 2) show that DM and DNM are positively and closely correlated, and together with SAM and GPRE, they mainly influence grouping along the first component. The second component is affected by GSH, *β*-glucans, Cu(II), and Time. The factors are positively correlated and responsible for grouping samples representing trial E and partly G. The third component (not shown) is influenced by the Ag(I) factor.

**Table 4:** The PCA factor loadings

	Components		
Factors	F1	F2	F3
Cu(II)	-0.133	**0.662**	-0.279
Ag(I)	0.310	0.020	**0.839**
Time	-0.482	-0.201	0.392
DNM	**0.907**	0.307	0.050
DM	**0.908**	0.239	0.067
SAM	**0.548**	-0.199	-0.484
GSH	-0.401	**0.743**	-0.096
*β*-glucan	-0.502	**0.681**	0.106
GPRE	0.362	**0.658**	0.180

**Fig. 3:**
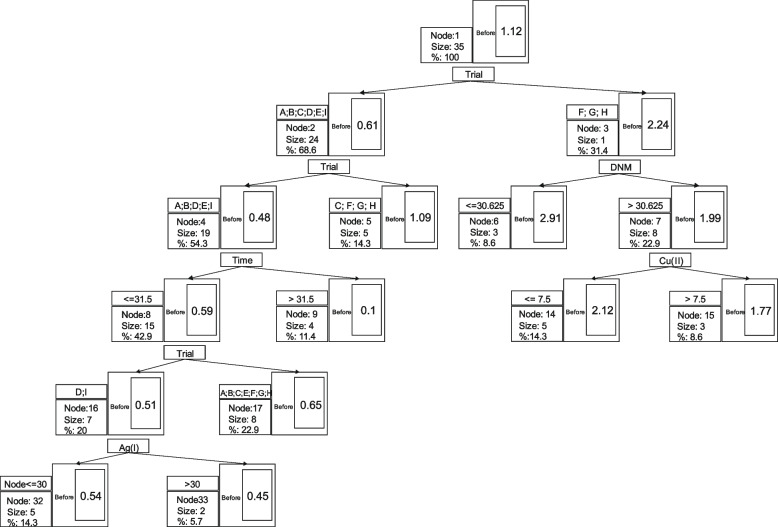
Classification and regression tree analysis illustrating trials classification

The classification and regression tree analysis (Fig. 3) shows that trials are the most influential factors for the first grouping level, with GPRE as a dependent variable. The left node, reflecting the second level, uses trials for further classification. Then, the time of anther cultures is crucial at the fourth level. Trials determine classification at the fifth level and silver ions at the sixth level. The second level classification (right node including F, G, and H trials) is due to DNM. The last classification concerning samples with DNM greater than 30.625 is due to Cu(II) ion concentration.

The curves for DNM, DM, SAM, GSH, *β*-glucans, and GPRE show evident changes related to trials. The E trial had the lowest GPRE, DNM, DM, and SAM readings. On the other hand, trial G yielded the greatest GPRE value, which matched the best GSH value. The DM value also outperformed the DNM value.

**Fig. 4:**
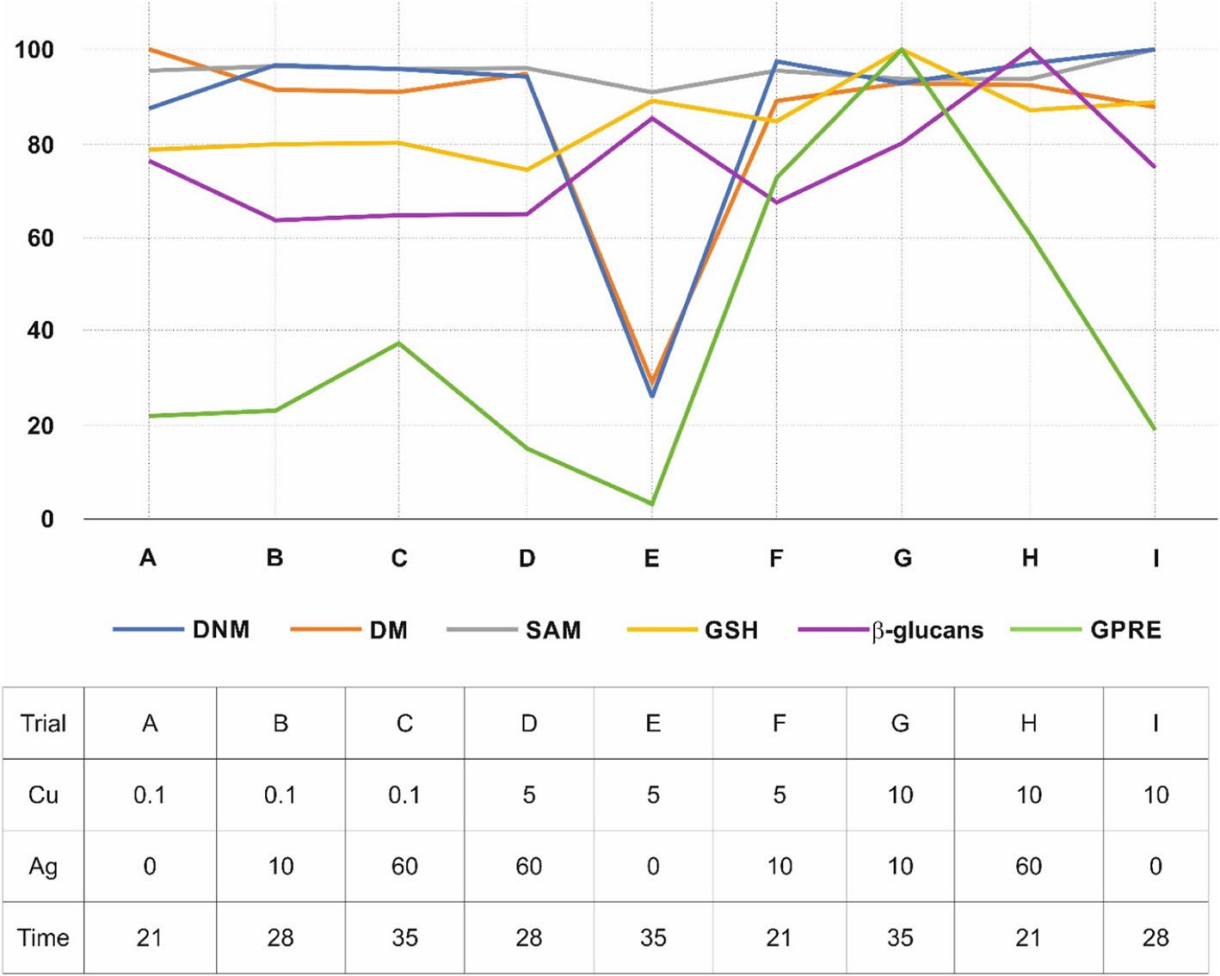
Standardized characteristics of DM, DNM, SAM, GSH, *β*-glucans, and GPRE for trials

The comparisons of the curves (Fig. 4) conducted via the Passig-Bablok regression analysis (Table 5) show curves of DM and DNM, DM and SAM, DM and *β*-glucans, SAM and GSH, SAM and *β*-glucans, GSH and *β*-glucans passed linearity of variables and were different. The relationship between DNM and SAM, DNM and GSH, DNM and *β*-glucans, DNM and GPRE, DM and GSH, SAM and GPRE, GSH and GPRE, and *β*-glucans and GPRE was not linear, whereas the model was significant, suggesting differences in curves.

**Table 5:**
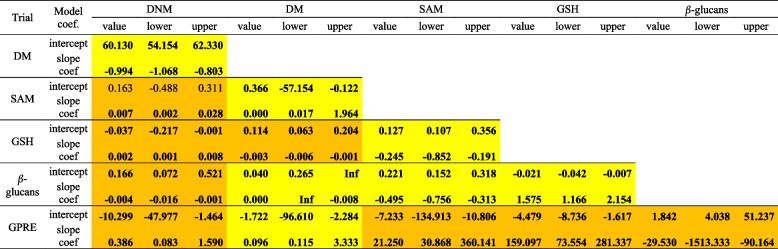
The Passing Bablok regression results indicating similarity between graphs regarding DNM, DM, SAM, GSH, *β*-glucans, and GPRE

Yellow cells – linearity of variables is assumed; orange cells – linearity of variables is not assumed; characters in green indicate that the given parameter is insignificant; characters in black shows significant model parameters

## Discussion

The study was based on a biological system [16] with varying anther culture conditions, involving a sophisticated molecular marker system (DArTseqMet) [35, 62] for methylome analysis, spectroscopic method (ATR-FTIR) [34] for analysis of variation in biochemical profile, and in putative compounds possibly related to GPRE.

The biological system encompasses barley donor plants, the generative progeny of a doubled haploid obtained by anther culture through microspore embryogenesis (androgenesis). Utilization of a single homozygous donor plant was dictated by the necessity of elimination of putative somaclonal [16] and pre-existing [63] variations that might impact regenerants. Preliminary verification of plant uniformity was tested at the morphological level. Plant height, leaf size, tillering, and seed set evaluated between donor plants and their regenerants derived via several experimental trials encompassing different concentrations of Cu(II), Ag(I), and time of anther culture revealed a lack of morphological differences among analyzed materials. It is a common fact that morphological differences between regenerants may not be very often detected [33] and, if necessary, could be easily eliminated during subsequent phases of anther culture plant regeneration. Thus, their lack could be addressed by a limited sample size or biological system employed to prevent putative variation sources [64]. Still, the lack of such differences does not exclude biochemical [34] or DNA methylation-related changes [31].

The presented work focuses on three substances of importance for obtaining green regenerants by tissue culture, including SAM, GSH, and *β*-glucans. SAM delivers a methyl group to various molecules, including proteins, lipids, hormones and nucleic acids [65], in methylation reactions catalyzed by numerous methyltransferases [66]. The imposition of a methyl group influences the regulation of gene expression, affects the mobility of membrane receptors and maintains membrane fluidity [67], in addition to affecting processes such as transsulfuration [68] and the production of ethylene [69] and polyamines [70]. SAM was shown to influence *de novo* methylation in triticale in plant tissue cultures, while *de novo* methylation was involved in GPRE [51]. The second compound is GSH, a small intracellular thiol molecule that is a potent non-enzymatic oxidant. Among its many functions in plant cells, glutathione prevents the oxidative denaturation of proteins under stress conditions by protecting their thiol groups [71, 72], and it is a substrate for glutathione peroxidase and glutathione S-transferase. The primary function of glutathione is its role in conferring tolerance to abiotic stress [73] and hence its applications in obtaining plants *by in vitro* culture [74, 75]. Finally, *β*-glucans are important for glycolysis [76, 77]. Glycolysis, which uses *β*-glucans, pumps the Krebs cycle, producing ATP [78]. Thus, the Krebs cycle is crucial for SAM and GSH as producing both chemical compounds is an ATP-dependent process [69, 79, 80].

In contrast, ATP synthesis requires copper ions as cofactors involved in elements of the respiratory chain [81]. The ATR-FTIR study employed leaves of young regenerants to analyze changes in biochemical compounds possibly reflecting the Krebs’, Yung, and GSH-ascorbate cycles. In this study we used the 1^st^ derivative [82–84] which provided enhanced resolution (Fig. 1) compared to raw spectra and thus facilitated the proper location of poorly resolved components in the complex spectrum of the plant material. The selected SAM, GSH, and *β*-glucans spectral regions proved to vary between trials, confirming that tissue culture conditions may affect the biochemical level of cells and that the influence is “remembered” in leave tissue during multiple rounds of cell deviations and tissue specification.

Based on triticale experiments [36] and the role of SAM [85], we have shown that SAM impacted DNA methylation what might result in gene expression changes affecting GPRE [51]. The DNA methylation changes were studied via DArTseqMet markers quantified with MSAP [58] semi-quantitative approach and converted into DMV and DM. The analysis revealed the presence of differences at the DNA methylation levels evaluated between regenerants regarding Cu(II), and Ag(I) ion concentrations in the IM and time of anther cultures. Thus, varying experimental conditions affecting microspore biochemistry, DNA methylation and GPRE should result in different sample groupings representing trials.

Unsurprisingly, the notion was confirmed by the PCoA analysis. Samples of some trials formed tight groups, and group formation was linked to the factors contributing to the two main components responsible for 55.05% of the variance. The most correlated factors were DNM, DM, and SAM, which had the most significant input for the first component. The second component was composed mainly of GSH, *β*-glucans, Cu(II), and GPRE that were moderately correlated (GSH to *β*-glucans and Cu(II) to GPRE). The Ag(I) and time had little input into the first two components, suggesting their limited role in barley anther culture plant regeneration. The fact was in agreement with structural equation modeling involving Cu(II), Ag(I), Time, DNM, DM, and GPRE, where the Ag(I) effect was identified but was weak [86].

While PCoA shows the input of loading factors to the sample classification, employing classification and regression analysis illustrates their hierarchy. Thus, the primary samples’ grouping was due to trials reflecting the *in vitro* tissue culture conditions used for plant regeneration. The trend is partly reflected at the second classification level, where 24 out of 35 samples were subdivided according to trials and the remaining 11 samples according to DNM. Time and Cu(II) were responsible for further classifications, whereas Ag(I) showed the lowest input, which is in agreement with PCoA.

The most straightforward result is possibly showing how *in vitro* tissue culture conditions affect biochemical levels, DNA methylation changes and GPRE. Depending on trials, the biochemical, DNA methylation levels change (and profiles of changes for the analyzed characteristics differ from each other as indicated by Passing Bablok regression analysis) with the most prominent fluctuation in the trial E and G. Low Cu(II) concentration, lack of Ag(I) in the medium and prolongated time of *in vitro* cultures exhibits decreased SAM level, somewhat increased GSH and *β*-glucans. At the same time, apparent depletion of DNM and DM is observed, leading to the lowest value of GPRE. Possibly, under such conditions, the Krebs cycle is not functioning efficiently, which is reflected by lowered SAM values, which results in DNA demethylation and decreased DNA *de novo* methylation. Although not studied, it sounds reasonable to postulate that gene expression (possibly poorly coordinated due to decreased DNA methylation level) is less directed towards plant regeneration or is not effectively regulated. Increased GSH may suggest oxidative stress. In parallel to low Cu(II) concentration, which is under elevated concentration, might reduce oxidative stress, it is becoming clear why GPRE achieved the lowest value among all trials tested.

On the contrary, the highest Cu(II), intermediate Ag(I) concentrations and prolongated time of other cultures resulted in the best values of GPRE. Cu(II) plays a crucial role in antioxidative stress, and its amount is sufficient for proper biochemical cycle functioning. Furthermore, the highest level of GSH supports the hypothesis that antioxidative components of the cell function at the highest levels, whereas high SAM values suggest that epigenetic control of gene expression may be well established. Moreover, DNM and DM are in balance, indicating the correct transcriptome functioning, which translates to increased GPRE.

## Conclusions

Our study shows complex interplay between biochemical and methylome levels affecting GPRE due to *in vitro* anther culture conditions and illustrates that varying the conditions one may affect plant regeneration. Furthermore, the observed changes affecting biochemical and DNA methylation levels seem congruent with the structural equation model presented for triticale [33], indicating that comparable mechanisms of plant regeneration via anther cultures may be involved in both cases. Although indicative, our results must be subjected to structural equation modelling analysis to draw the most significant relationships between analyzed factors. Still, increasing Cu(II) concentration in the IM affected metabolome and DNA methylation, raising GPRE. Thus, manipulating Cu(II) concentration in the IM is reasonable to suggest that it would benefit GPRE.

## Data Availability

The datasets used and/or analysed during the current study are available from the corresponding author on reasonable request.

## Abbreviations

2,4-D: 2,4-Dichlorophenoxyacetic acid
AFLP: Amplified Fragment Length Polymorphism
ATR: Attenuated Total Reflectance
ATR-FTIR: Attenuated Total Reflectance-Fourier Transform Infrared
DArTseqMet: Diversity Arrays Technology Methylation Analysis
DM: Demethylation
DNM: *de novo* Methylation
GPRE: Green plant regeneration efficiency
GSH: Glutathione
IAA: Indole-3-acetic acid
IM: Induction media
IRAP: Inter-Retrotransposon Amplified Polymorphism
ISSR: Inter-Simple Sequence Repeat
MSAP: Methylation-Sensitive Amplified Polymorphism
NAA: Naphthaleneacetic acid
RAPD: Random Amplified Polymorphic DNA
RFLP: Restriction Fragment Length Polymorphism
SAM: S-adenosyl-L-methionine
SSR: Simple Sequence Repeats
TCIV: Tissue culture-induced variation
